# Safety of Ready-to-Eat Green Leafy Salads: Growth Potential of *Listeria monocytogenes* During Shelf Life

**DOI:** 10.3390/foods15071136

**Published:** 2026-03-25

**Authors:** Muhammad-Ehtesham Abdul, Paolo Cipriani, Elena Cosciani-Cunico, Paola Monastero, Stefania Ducoli, Alessandro Norton, Daniela Merigo, Enrico Pavoni, Guido Finazzi, Marina-Nadia Losio, Elena Dalzini

**Affiliations:** 1 Istituto Zooprofilattico Sperimentale della Lombardia e dell’Emilia-Romagna “Bruno Ubertini”, 25124 Brescia, Italy; 2 Civil, Environmental, Architectural Engineering, and Mathematics (DICATAM), University of Brescia, 25121 Brescia, Italy

**Keywords:** minimally processed vegetables, foodborne pathogens, challenge test, food safety

## Abstract

Ready-to-eat (RTE) fresh salads are widely consumed for their convenience and nutritional value, but they could represent a relevant food safety concern, as they do not undergo a lethal heat treatment before consumption, and furthermore, they may support the growth of *Listeria monocytogenes* during refrigerated storage. In this study, the growth potential of *L. monocytogenes* was evaluated by standardised challenge tests in five commercially available RTE salads: crispy lettuce, baby lettuce, a baby lettuce–spicy mustard mix, and two mâche products from different producers. Three different batches for each product were inoculated with a three-strain cocktail of *L. monocytogenes* at a target level of approximately 2–3 log CFU/g and stored under conditions simulating reasonably foreseeable refrigerated storage (7 °C for approximately two-thirds of their shelf life, followed by 10 °C for the remaining one-third), in accordance with ISO 20976-1 and EURL *L. monocytogenes* guidelines. The growth potential (Δ) was calculated as the difference between the highest mean *L. monocytogenes* concentration observed during storage and the mean of the initial concentration at time zero, both in three replicate samples; Δmax was defined as the highest Δ value among the tested batches. Crispy lettuce, baby lettuce, and the mixed salad supported the growth of *L. monocytogenes*, with Δmax values of 2.33, 2.60, and 3.65 log CFU/g, respectively. In contrast, both mâche products showed Δmax values ≤ 0.5 log CFU/g, indicating an inability to support pathogen growth under the tested conditions. These results demonstrate that the growth potential of *L. monocytogenes* in RTE salads is strongly product-specific and likely influenced by intrinsic characteristics and background microbiota, as well as by storage temperature. The findings underline the importance of strict temperature control and product-specific risk assessment to ensure compliance with microbiological criteria throughout shelf life and to mitigate the risk of listeriosis associated with RTE salads.

## 1. Introduction

The consumption of ready-to-eat (RTE) fresh salads has increased markedly in recent years due to changing lifestyles, consumer demand for convenience, and the perception of fresh vegetables as healthy food choices [1]. Minimally processed leafy greens, including fresh-cut and mixed salads, are widely used in daily meals and catering services, but their production requires careful control to preserve sensory quality, nutritional value, and microbiological safety throughout shelf life [2,3,4,5].

Among foodborne pathogens, *L. monocytogenes* represents a major concern for RTE fresh produce. Unlike foods that undergo a lethal heat treatment, fresh-cut salads are consumed without further processing and may become contaminated at multiple points along the production chain, including primary production, washing, cutting, packaging, and distribution [6]. Several outbreaks and sporadic cases of listeriosis have been associated with the consumption of fresh and minimally processed vegetables, highlighting the public health relevance of this pathogen in plant-based foods [7]. Recent European data show an upward trend in invasive listeriosis. In 2024, 3041 confirmed cases were reported across 26 EU Member States (0.69 per 100,000 population), with 301 fatalities, predominantly among older adults [8].

RTE salads are considered high-risk matrices for *L. monocytogenes* due to a combination of intrinsic and extrinsic factors. The cutting and handling operations cause tissue damage, releasing nutrients and increasing surface moisture, which may favour bacterial attachment and growth [9,10]. In addition, these products typically exhibit pH and water activity values that are permissive for *L. monocytogenes* growth, while refrigerated storage conditions may not be sufficient to prevent its proliferation, particularly under temperature abuse scenarios [11,12]. The interaction with indigenous microbiota further complicates the prediction of pathogen behaviour, as background microflora may either inhibit or, in some cases, support *L. monocytogenes* survival and growth depending on product-specific characteristics [13,14]. Moreover, packaging conditions, including modified atmosphere packaging (MAP), can strongly influence microbial growth and should be considered when evaluating pathogen behaviour in RTE salads [15].

In the European Union, microbiological safety of RTE foods is regulated by Commission Regulation (EC) No 2073/2005, which establishes a limit of 100 CFU/g for *L. monocytogenes* throughout shelf life for foods that support its growth. Compliance with this criterion requires food business operators (FBOs) to demonstrate, through scientific evidence such as challenge tests or durability studies, that the pathogen will not exceed the regulatory limit under reasonably foreseeable storage conditions [16,17]. Comparable risk-based approaches requiring scientific evidence to demonstrate the safety of RTE foods are also applied in several non-EU countries, although specific regulatory limits and validation procedures may differ [18]. Recent regulatory updates have further strengthened the responsibility of food business operators along the entire distribution chain, including retail, emphasising the need for product-specific risk assessment and shelf-life validation. If the FBO cannot provide this information, then the limit of absence of 25 g must be applied during the shelf life [16,19].

Regarding the shelf life, it is also necessary to consider temperature as an important factor for pathogen growth. For this purpose, the European Union Reference Laboratory for *L. monocytogenes* (EURL Lm), Paris, France, launched an inquiry into its National Reference Laboratory network and reviewed the scientific literature from 2002 to 2020. The outcomes were integrated into the EURL Lm Technical Guidance Document to assess the shelf life of refrigerated RTE foods, which resulted in the recommendation to use 7 and 10 °C as the reference temperature to simulate the reasonably foreseen storage conditions in domestic refrigerators [20].

Despite the growing body of literature on *L. monocytogenes* in fresh produce, comparative data obtained under standardised challenge-test conditions across different commercially available RTE leafy salads remain limited. Previous studies often differ in plant species investigated, processing practices, packaging conditions, background microbiota, and experimental protocols, which makes cross-study comparisons difficult and limits the possibility of drawing product-specific conclusions.

In particular, few studies have evaluated multiple RTE salad products using the same standardised methodology recommended by the EURL* L. monocytogenes* guidance, which is necessary to reliably compare growth potential among products.

Therefore, this study aimed to evaluate the growth potential of *L. monocytogenes* in five commercially available RTE salads using standardised challenge tests conducted under conditions simulating reasonably foreseeable refrigerated storage. We hypothesised that the growth potential of *L. monocytogenes* would differ significantly among salad products, reflecting product-specific characteristics of the plant matrix and storage environment. By assessing pathogen behaviour throughout the assigned shelf life, this work seeks to contribute to product-specific risk evaluation and support evidence-based shelf-life determination for RTE fresh salads.

## 2. Materials and Methods

### 2.1. Standard Method

All challenge tests performed in this study were conducted in accordance with the ISO 20976-1 [21] document *“Microbiology of the food chain—Requirements and guidelines for conducting challenge tests of food and feed products—Part 1: Challenge tests to study growth potential, lag time and maximum growth rate”* and in compliance with the European Guidelines described in the *Technical guidance document on Challenge Tests and Durability Studies for Assessing Shelf-Life of Ready-to-Eat Foods Related to Listeria Monocytogenes* [20].

### 2.2. Strains Used

To account for the growth variability of *L. monocytogenes*, the products were inoculated with a multi-strain cocktail combining both reference and field strains. The mixture included *L. monocytogenes* ATCC^®^ 19115™ as a reference strain, *L. monocytogenes* 12MOB052LM supplied by the EURL, and *L. monocytogenes* 253565/2006, a strain isolated in the IZSLER laboratory from a RTE leafy salad (fresh-cut lettuce mix), representative of the matrices investigated in this study. This multi-strain approach was employed to better represent the potential variability in the growth behaviour of *L. monocytogenes* under the experimental conditions.

### 2.3. Inoculum Preparation

The *L. monocytogenes* strains, stored at –80 °C, were individually cultured in Brain Heart Infusion (BHI) broth (Oxoid Italia, Milan, Italy) and incubated at 37 °C for 15–18 h, until reaching the early stationary phase. Each strain was then subcultured in BHI and incubated at 7 °C for 7 days to allow adaptation to the refrigerated conditions representative of the product’s storage during the shelf-life study. Before preparation of the inoculum cocktail, the concentration of each strain was individually determined by plate counting on Agar Listeria Ottaviani Agosti (ALOA) agar plates (Biolife Italiana Srl, Milan, Italy). Cell suspensions were then adjusted to the same target concentration by dilution in sterile buffered peptone water. Equal volumes of each standardised suspension were combined to obtain a mixed inoculum, ensuring that each strain contributed equally to the final concentration. The target inoculum level was approximately 2–3 log CFU/g for all tested products. This level was intentionally selected because RTE leafy vegetables generally can harbour indigenous microbial populations, which may interfere with the enumeration and growth dynamics of *L. monocytogenes*, as recommended by international guidelines and supported by previous studies on fresh produce [20,21,22].

### 2.4. RTE Fresh Salads

Five types of RTE salad were provided by local companies just after the packaging, at the beginning of the shelf life, and transported to IZSLER’s laboratory in refrigerated conditions (+4 ± 2 °C). Three different batches were used for each RTE salad to perform the challenge test. The RTE salads were Crispy lettuce consisting of ready-to-eat lettuce (*Lactuca sativa*) with an assigned shelf life of 12 days; Mixed salad containing green baby lettuce (85%) (*Lactuca sativa* var. *acephala*) and red mustard with wasabi (15%) (*Brassica juncea* var. *crispa* and *Brassica juncea* var. *wasabina*), with an assigned shelf life of 14 days; Mâche (A) (*Valeriana* spp.) with an assigned shelf life of 6 days, whereas Mâche (B) of the same species has a longer shelf life (9 days); and Baby lettuce (*Lactuca sativa* var*. acephala*) with an assigned shelf life of 6 days.

The difference in the assigned shelf life between Mâche (A) and Mâche (B) reflects the fact that these are products from two different suppliers, with potentially different production practices and initial quality parameters.

### 2.5. Units Preparation and Inoculation

For each product, single units (single salad packages) were prepared according to EURL Lm guidelines [20]. For each batch, a total of 33 units were allocated, divided into three experimental groups:

1. Food control units—units without manipulation (9 units)
2. Control units-inoculated with sterile physiological solution (1% *v*/*w*) (9 units)
3. Test units—inoculated with *L. monocytogenes* suspension (1% *v*/*w*) (15 units)

The inoculation of control units and test units was performed using a sterile graduated syringe through a septum set on the product’s packaging. After inoculation, a second septum was applied to cover the first, ensuring package integrity. Although the septum-based inoculation method was used to minimise disturbance of the original package atmosphere, the gas composition (O_2_ and CO_2_) inside the packages was not directly measured during the study. Then, the leaves were gently shaken to ensure even spread of the inoculum while maintaining the integrity of the original package atmosphere and minimising mechanical damage. In addition, BHI broth was inoculated with the same *L. monocytogenes* suspension and incubated under the same temperature regimes as the products to provide a positive control aimed at confirming the strains’ ability to grow under the defined experimental temperature conditions, without interference from the food matrix. All the units were immediately stored under temperature conditions simulating the most probable storage scenarios until consumption, in accordance with EURL Lm guidance [20]. The specific incubation regimes for each product were as follows:

○ Crispy lettuce: 7 °C for 8 days, then 10 °C for 4 days
○ Mixed salad: 7 °C for 9 days, then 10 °C for 5 days
○ Mâche (A): 7 °C for 4 days, then 10 °C for 2 days
○ Mâche (B): 7 °C for 6 days, then 10 °C for 3 days
○ Baby lettuce: 7 °C for 4 days, then 10 °C for 2 days

The selected temperature profile was chosen to simulate reasonably foreseeable refrigeration conditions along the cold chain. The EURL Lm Technical Guidance Document recommends using a higher temperature (7 °C) to reflect chill chain storage and distribution and a moderate temperature increase (10 °C) to represent likely domestic refrigerator abuse, based on observed consumer storage data. This approach ensures that growth potential assessments account for realistic storage variability

### 2.6. Microbial and Physicochemical Analyses

Approximately 25 g of each sample was transferred into a one-chamber filter stomacher bag (Neomed, Milano, Italy) and homogenised 1:10 (*w*:*v*) in Buffered Peptone Water (BPW) (Biolife Italiana srl, Milan, Italy) for 2 min using a Stomacher 400 blender (Seward Medical, London, UK). Decimal dilutions in sterile BPW were then prepared. For the positive control in broth, 1 mL of inoculum was directly diluted in BPW.

For each batch, at the beginning and end of storage, both in the control and food control units (not contaminated units), the total viable count (TVC) was done according to ISO 4833-1 [23] in Plate Count Agar (PCA, Biolife, Milan, Italy), and the plates were incubated at 30 °C for 72 h. *Enterobacteriaceae* were enumerated according to ISO 21528-2 [24], using Violet Red Bile Glucose Agar (VRBG, Thermo Fisher Scientific, Milan, Italy), and plates were incubated at 30 °C for 48 h. Additionally, lactic acid bacteria (LAB) were enumerated by pour plating 1 mL of the appropriate dilution in de Man, Rogosa, and Sharpe Agar (MRSA, Microbiol Diagnostici, Cagliari, Italy) and incubating according to ISO 15214 [25]. The pH of the samples was measured on 10 g of product after homogenization of the leaves to obtain a uniform slurry, using an HI 223 Calibration Check Microprocessor pH meter (Hanna Instruments, Smithfield, RI, USA) equipped with a Gel-Glass electrode (Hamilton, Bonaduz AG, Switzerland). Water activity (a_w_) was determined at 25 °C using an AquaLab series 3 TE recorder (Decagon Devices, Inc., Pullman, WA, USA), following ISO 18787 [26]. The storage temperature was monitored and recorded in one control and test unit per batch using a Thermo Button 22 L data logger (Astori Tecnica s.n.c., Poncarale, Brescia, Italy). In test units (contaminated units), the enumeration of *L. monocytogenes* was performed according to ISO 11290-2 [27]. Typical colonies were counted after incubation of duplicate plates of ALOA agar at 37 °C for 48 or 72 h. Upon arrival at the laboratory, detection of *L. monocytogenes* was also performed in a food control sample of each batch according to ISO 11290-1 [28] to verify the absence of natural contamination.

Microbial and physicochemical analyses on non-contaminated samples were performed on three units (independent biological replicates) at the beginning and at the end of the shelf life, while only one unit was analysed at each intermediate sampling point. For contaminated samples, three units per group were analysed at each of the five sampling points throughout the shelf life (Table 1). The selection of sampling intervals was designed to ensure that at least five sampling points (including t_0_) were obtained over the shelf life of each product. The intervals were therefore not uniform across products, reflecting differences in the assigned shelf life in line with ISO 20976-1 recommendations for challenge testing [21], while maintaining experimental feasibility.

### 2.7. Data Analysis

Microbiological counts, expressed in CFU/g, were converted to log CFU/g for the data analysis. For each RTE salad, the mean value and standard deviation were calculated at each sampling point, separately for the three tested batches. The growth potential (Δ) of *L. monocytogenes* was then calculated for each batch as the difference between the highest mean concentration observed during the shelf life (log_max_) and the mean concentrations measured at the beginning of the shelf life (log_i_) [21]. The maximum growth potential (Δmax) was determined as the highest Δ value observed among the three tested batches.

According to the criteria of this study, a product was considered able to support the growth of *L. monocytogenes* if Δmax exceeded 0.5 log. Conversely, if Δmax was equal to or lower than 0.5 log CFU/g, the product was presumed unable to support the growth of the pathogen. To compare growth potential (Δ) among the five salad products, a one-way analysis of variance (ANOVA) was performed using the Δ values calculated for each independent production batch, resulting in three observations per product (*n* = 3), with product as the fixed factor. The assumptions of ANOVA, including normality of residuals and homogeneity of variances, were verified using the Shapiro–Wilk and Levene’s tests, respectively. When a significant effect of product on growth potential was detected, Tukey’s Honest Significant Difference (HSD) post hoc test was applied to identify pairwise differences among products [29,30,31,32]. All statistical tests were two-tailed and performed using R version 4.5.2, with significance set at *p* < 0.05. In not-contaminated products, the indigenous microorganisms, pH and a_w_ were measured on three sample units, and the results were reported as the mean and standard deviations for each sampling point (t_0_ and t_end_) for three batches. Student’s *t*-tests were used to compare control and food-control units and to evaluate changes in microbial counts during storage (t_0_ vs. t_end_) within each product. For each product, three packages per batch were analysed at the initial and final sampling points. The *t*-test was applied using the mean values calculated from the three independent production batches. If Student’s *t*-test indicated no significant differences between control units and food control units, the final concentrations of microbial populations measured in the control units were used for subsequent analyses.

Following this, an exploratory Spearman rank correlation analysis was performed on end-of-shelf-life microbiological data to qualitatively assess potential associations between background microbiota (LAB, TVC, and Enterobacteriaceae) and Δmax of *L. monocytogenes.* Due to the small number of products (n = 5), this analysis was considered exploratory and descriptive, and no inferential statistical tests or *p*-values were reported. This approach allowed assessment of trends and potential microbiota-pathogen interactions while avoiding overinterpretation of the limited dataset.

## 3. Results

Three separate batches of each RTE salad were analysed to evaluate microbiological and physicochemical changes during the shelf life. For each analysed parameter, no significant differences were observed between control and food control units (Student’s *t*-test, *p* > 0.05) at t_0_ and t_end_.

The initial levels of LAB were very low, below the quantification limit. Only in Baby Lettuce was it possible to detect an initial concentration of 2.25 ± 0.35 log CFU/g, which increased to 3.59 ± 0.33 log CFU/g by the end of the shelf life.

TVC at the beginning of the shelf life ranged from 2.64 ± 0.91 in Crispy Lettuce to 5.81 ± 0.32 in Mixed Salad, and a significant increment (*p* < 0.05) was observed during the storage of all products. For example, in Crispy Lettuce, TVC increased from 2.64 ± 0.91 log CFU/g at t_0_ to 5.54 ± 0.55 log CFU/g at t_end_ in control units, and from 2.79 ± 0.36 to 5.64 ± 0.37 log CFU/g in food control units. In Mixed Salad, TVC increased from 5.81 ± 0.32 log CFU/g at t_0_ to 8.22 ± 0.49 log CFU/g at t_end_ in control units, with similar trends in food control units (Table 2). The concentration of Enterobacteriaceae was not analysed in Crispy Lettuce during the whole shelf life, while in the control units of all the other products, the concentration ranged from 1.99 ± 0.53 log CFU/g in Mâche (A) to 3.77 ± 0.62 log CFU/g in Baby Lettuce, with a significant increase during shelf life (*p* < 0.05). A similar trend was also observed in the food-control units.

The pH values of all products tended to increase during storage. In Crispy Lettuce, the change was limited and not significant (from 5.9 ± 0.08 to 6.05 ± 0.13 in control units), whereas in Mixed salad, a significant increase (*p* < 0.05) was observed (from 5.92 ± 0.11 to 6.95 ± 0.31). Similarly, in Mâche and Baby Lettuce, pH values rose by approximately 0.3–0.7 units over the shelf life. Water activity remained relatively stable across all products, generally ranging from 0.98 to 0.99, with only minor and not significant fluctuations (Table 3).

**Figure 1:**
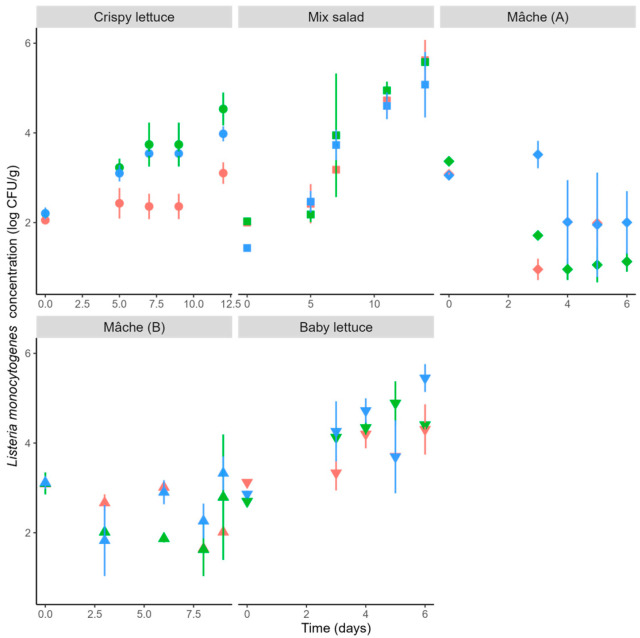
**Growth dynamics of *****L. monocytogenes***** in five RTE salads during refrigerated storage.** Symbols and error bars represent mean concentrations and standard deviations calculated from three sample units at each sampling point, across three independent batches (orange = batch 1, light blue = batch 2, green = batch 3).

**Figure 2:**
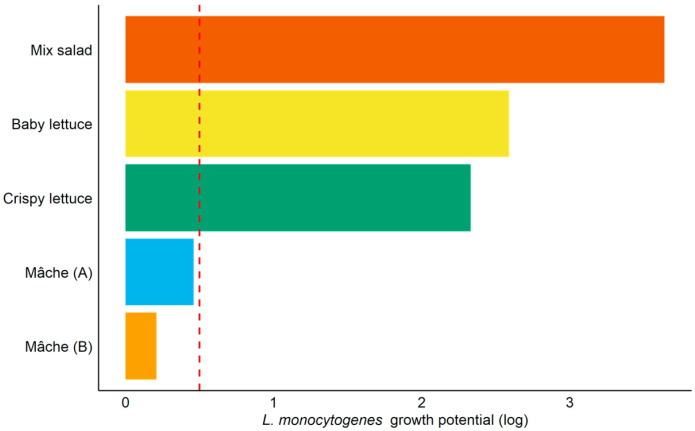
**Maximum growth potential (Δmax) of *****L. monocytogenes***** in five RTE salads.** Bars represent the highest Δ value observed among the three tested batches for each product. The red dashed line indicates the 0.5 log CFU/g threshold, above which a product is considered to support the growth of *L. monocytogenes*.

Initial inoculum levels of *L. monocytogenes* in the tested batches ranged from 1.43 to 3.12 log CFU/g. These differences reflect routine practical variability during inoculation and handling and do not affect the assessment of growth potential (Δ and Δmax). *L. monocytogenes* growth was observed during the storage period in all products except both Mâche types (Figure 1), which showed maximum growth potentials (Δmax) of 0.47 and 0.21 log CFU/g, respectively. According to the criteria established in this study (Δmax ≤ 0.5 log CFU/g), these two products did not support the growth of *L. monocytogenes*, whereas the other salads allowed substantial growth, with log_max_ values ranging from 3.10 to 5.62 log CFU/g (Figure 2). The growth potential, calculated as the difference between log_max_ and log_i_ for each batch, ranged from 0.21 to 3.65 log CFU/g (Table 4).

Assumptions for ANOVA were verified before hypothesis testing: the Shapiro–Wilk test indicated that residuals were normally distributed, and Levene’s test confirmed homogeneity of variances among products, supporting the use of a standard one-way ANOVA. A one-way ANOVA revealed a highly significant effect of product on the growth potential (Δ) of *L. monocytogenes* ((F/4,10) = 31.3, *p* < 0.05). Effect size, calculated as eta-squared (η^2^ = 0.93), indicates that the majority of variability in growth potential is explained by product type. Detailed ANOVA results and 95% confidence intervals for each product are reported in Supplementary Tables S1 and S2. Post hoc comparisons using Tukey’s HSD showed that Mixed salad had a significantly higher growth potential than all other products (adjusted *p* < 0.05). Baby Lettuce and Crispy Lettuce exhibited intermediate growth potential values, each significantly greater than Mâche (A) and Mâche (B) (adjusted *p* < 0.05), but lower than Mixed salad. No significant difference was observed between Mâche (A) and Mâche (B) (adjusted *p* ≈ 0.999), indicating similar growth potential in these two products. Detailed Tukey post hoc results are provided in Supplementary Table S3. As expected, no single product reached concentrations comparable to those observed in the positive control broth, where the maximum *L. monocytogenes* concentration was approximately 9 log CFU/g. These results indicate systematic differences in *L. monocytogenes* growth potential across salad types, with Mixed salad exhibiting the greatest increase during shelf life.

An exploratory Spearman rank correlation analysis was conducted to assess potential associations between end-of-shelf-life microbiota (LAB, TVC, and Enterobacteriaceae) and the maximum growth potential (Δmax) of *L. monocytogenes.* Since no significant differences were observed between control units and food control units (Student’s *t*-test, *p* > 0.05), the final concentrations of microbial populations from control units were used for this analysis. Due to the limited number of products (n = 5), this correlation analysis was considered descriptive and exploratory. The Spearman correlation coefficients (ρ) suggested a negative association between LAB concentrations and Δmax (ρ ≈ −0.7), with higher LAB levels corresponding to lower pathogen growth potential. Enterobacteriaceae showed a positive association with Δmax (ρ ≈ 0.6), whereas TVC displayed no clear trend (ρ ≈ 0.2). These observations indicate that the levels of indigenous microbial groups, rather than total bacterial load alone, may influence the permissiveness of the RTE salad matrix for *L. monocytogenes* proliferation. Given the small sample size, these results should be interpreted cautiously and considered hypothesis-generating.

## 4. Discussion

The growth potential of *L. monocytogenes* in RTE salads in this study was highly product-specific, probably reflecting the combined influence of indigenous microbial groups, leaf surface characteristics, intrinsic antimicrobial compounds, and storage conditions.

In this study, five types of RTE salads were studied during the shelf life at 7 and 10 °C to calculate the growth potential of *L. monocytogenes*.

In the present study, the LAB, TVC and Enterobacteriaceae concentrations were measured at the beginning and at the end of the shelf life. A low initial concentration of LAB (less than 2 to 2.55 log CFU/g) and Enterobacteriaceae (less than 2 to 3.92 log CFU/g) was found in the tested salad. Some members of these groups, due to their facultative anaerobic metabolism, may be able to adapt to low-oxygen environments, potentially increasing in number during the shelf life of the salads. The extent of growth, however, can vary depending on the specific strains and storage conditions.

Similar microbiological dynamics have been reported in fresh-cut leafy vegetables. Ortega-Sanz et al. [33] observed an initial low microbial concentration in fresh-cut iceberg lettuce, with a concentration of 1.6 ± 0.3 log CFU/g and 3.1 ± 0.3 log CFU/g for LAB and Enterbacteriaceae, respectively. Then, a growth above 7.5 log CFU/g by the end of the 12 days of shelf life was observed in this study. However, in contrast to that study, limited growth of LAB was observed, with a maximum concentration of 3.59–3.56 log CFU/g. Ziegler et al. reported similar increases in TVC and Enterobacteriaceae in RTE salads at 5–8 °C, with the highest growth observed in parsley and mixed salads [34]. Sant’Ana et al. reported initial aerobic mesophilic counts between 5.5 and 8.1 log CFU/g in various RTE vegetables, with further increases during storage, particularly under temperature abuse conditions [35]. Although absolute initial TVC values in our study were generally lower, the observed increases during storage are consistent with their findings, reinforcing the notion that RTE vegetables provide a favourable environment for microbial proliferation even under refrigeration. The relatively lower increase in LAB populations observed in our study may be one of several factors contributing to the limited inhibition of *L. monocytogenes* observed in some products. Although this study did not directly assess inhibitory metabolites, specific LAB taxa, or antagonistic activity, LAB have been associated with antimicrobial effects in RTE vegetables. Previous studies indicate that such effects are not systematic and likely depend on specific microbial taxa and their metabolic activity rather than total LAB counts alone [35]. In the present study, indigenous microbiota were not characterised at the species or strain level, limiting direct attribution of inhibitory mechanisms. Nevertheless, an exploratory analysis of end-of-shelf-life data suggested a negative association between LAB concentrations and the Δmax of *L. monocytogenes*, indicating a possible protective role of higher LAB levels. However, this relationship was not strictly linear across products.

All products exhibited high water activity (a_w_ ≈ 0.98–0.99), suitable for bacterial growth. pH increased slightly over time: from 5.9–6.32 at t_0_ to 6.05–6.77 at the end of shelf life. Mixed Salad showed the most pronounced pH increase (5.92 ± 0.11 to 6.95 ± 0.31), likely contributing to higher bacterial growth. Other salads, such as Mâche, exhibited smaller pH changes, consistent with their lower microbial proliferation. These observations are in agreement with Ziegler et al., who reported near-neutral pH and high a_w_ in salads that supported *L. monocytogenes* growth [34].

In this study, Mixed Salad, Baby Lettuce, and Crispy Lettuce supported substantial pathogen proliferation, with maximum growth potentials (Δmax) of 3.65, 2.60, and 2.33 log CFU/g, respectively, whereas both Mâche (A) and Mâche (B) exhibited Δmax values below 0.5 log CFU/g, indicating that these products did not support growth (Figure 2, Table 4).

These results align well with previous challenge test studies on RTE vegetables. Alegbeleye and Sant’Ana reported that the growth potential of *L. monocytogenes* in RTE salads varies widely across vegetable matrices. In their study, several leafy vegetables supported growth exceeding 2–3 log CFU/g under refrigerated or mildly abusive temperatures, comparable to the increases observed in Mixed salad and lettuce-based products in the present study [14,35]

Crucially, the vegetable type itself has been identified as a dominant factor influencing *L. monocytogenes* behaviour, sometimes outweighing the effects of packaging atmosphere or storage temperature [36].

Trends observed in our exploratory analysis suggest a potential influence of indigenous microbiota (LAB, TVC, Enterobacteriaceae) on *L. monocytogenes* growth. However, in this study, due to the limited number of products tested (n = 5), these correlations are strictly hypothesis-generating and cannot be interpreted as causal relationships. These hypotheses are consistent with previous studies showing that background microflora can either suppress or promote pathogen growth depending on microbial composition and the food matrix [37,38,39,40]. For example, the products exhibiting higher Enterobacteriaceae concentrations tended to show higher Δmax values, whereas products with lower Enterobacteriaceae loads, such as both Mâche samples, were associated with minimal or no *L. monocytogenes* growth. This suggests that Enterobacteriaceae levels may serve as an indirect indicator of an ecological niche permissive to pathogen proliferation. This aligns with previous studies in which food products with higher concentrations of Enterobacteriaceae also showed greater *L. monocytogenes* growth [41,42]. Mechanical damage to leaves from handling, washing, or inoculation can disrupt plant defence barriers, exposing intracellular nutrients and creating localised moisture retention that promotes bacterial proliferation [43,44], potentially explaining why salads such as Mixed salad and Baby lettuce exhibited higher growth compared to Mâche, where minimal leaf damage likely preserved surface integrity. Previous studies suggest that the lack of growth observed in Mâche may be influenced by factors beyond mechanical damage, although these were not measured in our study. Mâche has been reported to exhibit low attachment values for *Salmonella enterica*, attributed to leaf surface hydrophobicity and epicuticular wax composition. Although *L. monocytogenes* employs different adhesion mechanisms and generally binds more effectively to biotic and abiotic surfaces, the same leaf traits that limit *Salmonella* attachment may reduce initial *L. monocytogenes* adhesion, thereby restricting subsequent proliferation [45,46,47]. Furthermore, it can be hypothesised that the presence of compounds such as thymol, a phenolic molecule with known antimicrobial activity against a broad spectrum of pathogens, including *L. monocytogenes*, could contribute to limiting pathogen growth, even if this was not directly assessed in our study [45,48,49]. Similarly, previous reports of Listeria-specific inhibitory activity in carrots (attributed to 6-methoxymellein and polyacetylenes) and rocket salad (glucosinate-derived isothiocyanates) provide context, but these effects remain speculative in relation to Mâche [50,51].

Statistical analyses confirmed that RTE salad type significantly affected *L. monocytogenes proliferation*; assumptions for one-way ANOVA were verified with Shapiro–Wilk and Levene’s tests [29,30,31], and the results indicated a significant effect of product on growth potential (F(4,10) = 31.3, *p* < 0.001), with post hoc Tukey’s HSD showing that Mixed salad had a significantly higher Δ than all other products, Baby Lettuce and Crispy Lettuce had intermediate values, and both Mâche types showed minimal growth. However, given the limited number of independent observations per product, these differences should be interpreted cautiously.

It is important to note that in this study, the indigenous microbiota was not fully characterised at the species or strain level, and differences in microbial composition between salad types were not directly assessed. Future investigations focusing on the identification and functional roles of native microbial communities could provide valuable insights into their inhibitory or promotive effects on *L. monocytogenes* and help refine predictive models for pathogen growth in RTE salads.

In addition, the regulatory implications of these findings are highly relevant. According to Regulation (EC) 2073/2005, RTE foods are required to contain less than 100 CFU/g of *L. monocytogenes* throughout their shelf life. The observed Δmax values indicate that, for products such as Mixed Salad, Baby Lettuce, and Crispy Lettuce, even low initial contamination levels could exceed this threshold during shelf life. For instance, an initial contamination of 1 log CFU/g combined with a Δmax of 3.65 log CFU/g in Mixed Salad would result in approximately 4.65 log CFU/g at the end of shelf life, clearly surpassing the regulatory limit. This highlights the critical importance of controlling storage conditions, handling practices, and shelf life to ensure compliance. Conversely, products like Mâche, with minimal pathogen growth, present a lower microbiological risk and are more likely to remain compliant throughout shelf life. These findings emphasise that both product type and intrinsic growth potential must be considered in risk assessment and quality control strategies for RTE salads.

## 5. Conclusions

The results obtained in this study highlight that the growth potential of *L. monocytogenes* in ready-to-eat salads is highly product-specific. Among the tested products, Crispy Lettuce, Baby Lettuce, and Mixed Salad supported substantial pathogen growth (Δmax > 2 log CFU/g), whereas both Mâche (A) and Mâche (B) showed minimal growth (Δmax ≤ 0.5 log CFU/g). Consumer handling practices, including proper refrigeration and adherence to recommended shelf life, can further influence the risk of *L. monocytogenes* proliferation. These findings indicate that strict temperature control during storage and distribution is important to manage potential pathogen growth, particularly in products that show higher growth in our study.

These observations are consistent with previous reports of substantial growth in lettuce varieties stored under mild temperature abuse and limited proliferation in products with antimicrobial properties or leaf traits that reduce attachment [13,29,31,49,50,51]. While these studies provide context, our study did not directly measure antimicrobial compounds, leaf surface characteristics, or bacterial attachment.

Overall, the results underscore the importance of product-specific assessments for RTE salads to ensure compliance with European regulations (EC 2073/2005), as refrigeration up to 10 °C may allow significant pathogen growth in some products while others remain more stable under the same conditions.

## Figures and Tables

**Table 1:** Sampling time considered during the challenge test in the five RTE salads used in this study, starting from the beginning of the shelf life (t_0_) to the end (t_end_) and including three additional points (t_1_, t_2_ and t_3_) for the sampling of contaminated test units.

RTE Salads	Sampling Time (Days)				
	t_0_	t_1_	t_2_	t_3_	t_end_
Crispy Lettuce	0	5	7	9	12
Mixed Salad	0	5	7	11	14
Mâche (A)	0	3	4	5	6
Mâche (B)	0	3	6	8	9
Baby Lettuce	0	3	4	5	6

**Table 2:** LAB, TVC, and Enterobacteriaceae concentrations (log CFU/g) in control and food control units of RTE salads at the beginning (t_0_) and at the end (t_end_) of the shelf life. Values are expressed as mean ± standard deviation of three replicates for three batches. NA = not analysed. The notation “<2” for concentrations at t_0_ corresponds to the detection limit of the enumeration method.

RTE Salads	Sample	LAB (log CFU/g)		TVC (log CFU/g)		Enterobacteriaceae (log CFU/g)	
		t_0_	t_end_	t_0_	t_end_	t_0_	t_end_
Crispy Lettuce	Control Unit	<2	2.53 ± 0.31	2.64 ± 0.91	5.54 ± 0.55	NA	NA
	Food Control Unit	<2	2.43 ± 1.31	2.79 ± 0.36	5.64 ± 0.37	NA	NA
Mixed salad	Control Unit	<2	1.90 ± 1.09	5.81 ± 0.32	8.22 ± 0.49	3.51 ± 0.25	7.09 ± 0.35
	Food Control Unit	<2	2.12 ± 1.40	5.79 ± 0.26	7.93 ± 0.68	3.55 ± 0.36	7.14 ± 0.29
Mâche (A)	Control Unit	<2	2.63 ± 0.05	5.33 ± 0.48	7.38 ± 0.47	1.99 ± 0.53	4.08 ± 1.25
	Food Control Unit	<2	2.82 ± 0.26	5.44 ± 0.93	7.46 ± 0.39	2.08 ± 0.39	4.20 ± 0.81
Mâche (B)	Control Unit	<2	3.18 ± 0.33	5.65 ± 0.24	8.03 ± 0.33	1.65 ± 0.39	4.60 ± 0.96
	Food Control Unit	<2	2.88 ± 0.40	5.60 ± 0.34	7.97 ± 0.46	3.59 ± 0.33	4.89 ± 0.89
Baby Lettuce	Control Unit	2.25 ± 0.35	3.59 ± 0.33	4.61 ± 0.70	8.53 ± 0.65	3.77 ± 0.62	6.83 ± 0.58
	Food Control Unit	2.55 ± 1.07	3.56 ± 0.20	5.57 ± 0.61	8.42 ± 0.59	3.92 ± 0.96	6.87 ± 0.50

**Table 3:** pH and water activity a_w_ values measured in control and food control units of RTE salads at the beginning (t_0_) and end (t_end_) of the shelf life. Values are expressed as mean ± standard deviation of three batches.

RTE Salads	Sample	pH		a_w_	
		t_0_	t_end_	t_0_	t_end_
Crispy Lettuce	Control Unit	5.90 ± 0.08	6.05 ± 0.13	0.99 ± 0.01	0.99 ± 0.01
	Food Control Unit	5.94 ± 0.04	6.03 ± 0.11	0.99 ± 0.00	0.99 ± 0.01
Mixed salad	Control Unit	5.92 ± 0.11	6.95 ± 0.31	0.99 ± 0.00	0.98 ± 0.00
	Food Control Unit	5.95 ± 0.13	6.80 ± 0.31	0.99 ± 0.00	0.99 ± 0.00
Mâche (A)	Control Unit	6.38 ± 0.18	6.60 ± 0.28	0.99 ± 0.00	0.98 ± 0.01
	Food Control Unit	6.41 ± 0.07	6.70 ± 0.15	0.99 ± 0.00	0.99 ± 0.00
Mâche (B)	Control Unit	6.41 ± 0.16	6.77 ± 0.22	0.99 ± 0.00	0.99 ± 0.00
	Food Control Unit	6.40 ± 0.11	6.72 ± 0.28	0.99 ± 0.00	0.99 ± 0.00
Baby Lettuce	Control Unit	6.32 ± 0.14	6.71 ± 0.16	0.99 ± 0.01	0.98 ± 0.01
	Food Control Unit	6.26 ± 0.11	6.77 ± 0.10	0.99 ± 0.01	0.98 ± 0.01

**Table 4:** Growth potential (Δ) and maximum growth potential (Δmax) of *L. monocytogenes* in RTE salad products stored at 7 °C and 10 °C Δ values were calculated for each batch as the difference between the highest mean concentration (log_max_) and the initial mean concentration (log_i_). The Δmax value represents the highest Δ detected among the three batches for each product.

RTE Salads	Batch	log_i_	log_max_	Growth Potential of *L. monocytogenes*	
				Δ	Δ max
Crispy Lettuce	1	2.05	3.10	1.05	2.33
	2	2.20	4.53	2.33	
	3	2.21	3.98	1.77	
Mixed salad	1	2.00	5.62	3.62	3.65
	2	2.03	5.58	3.55	
	3	1.43	5.08	3.65	
Mâche (A)	1	3.08	2.01	0.00	0.47
	2	3.37	1.71	0.00	
	3	3.05	3.52	0.47	
Mâche (B)	1	3.09	3.01	0.00	0.21
	2	3.10	2.79	0.00	
	3	3.12	3.33	0.21	
Baby Lettuce	1	3.12	4.30	1.18	2.60
	2	2.70	4.89	2.19	
	3	2.86	5.45	2.60	

## Data Availability

The original contributions presented in this study are included in the article. Further inquiries can be directed to the corresponding author.

## Supplementary Materials

The following supporting information can be downloaded at https://www.mdpi.com/article/10.3390/foods15071136/s1, Supplementary Table S1. Growth potential of *L. monocytogenes* (Δ) in RTE salads. Δ values are reported for each batch of the five tested products. The mean Δ, maximum Δ (Δmax), and 95% confidence intervals (CI) were calculated using three replicates per product. Δmax was defined as the highest Δ observed among the three batches. CI were calculated using the t-distribution. Supplementary Table S2. One-way ANOVA of growth potential (Δ) across five RTE salad products. The table reports degrees of freedom (Df), sum of squares (Sum Sq), mean squares (Mean Sq), F value, and *p*-value for the ANOVA model. A significant effect of product on growth potential was observed (F(4,10) = 31.3, *p* < 0.05). Supplementary Table S3. Pairwise comparisons of growth potential (Δ) among five RTE salad products using Tukey HSD. The table reports mean differences (Δ) between products, 95% confidence intervals (CI) for the differences, and adjusted *p*-values for multiple comparisons. Comparisons with adjusted *p* < 0.05 indicate statistically significant differences in growth potential.
