# Supplementary material for: Safety of Ready-to-Eat Green Leafy Salads: Growth Potential of Listeria monocytogenes During Shelf Life
Source: Foods. 2026 Mar 25;15(7):1136. doi: 10.3390/foods15071136 (PMC13072739; doi:10.3390/foods15071136)
Supplement: Supplementary file 1 [file foods-15-01136-s001.zip › Supplementary_Table_S1_Delta_CI.pdf]

Supplementary Table S1. Growth potential of *L. monocytogenes* (Δ) in RTE salads.

Δ values are reported for each batch of the five tested products. The mean Δ, maximum Δ (Δmax), and 95% confidence intervals (CI) were calculated using three replicates per product. Δmax was defined as the highest Δ observed among the three batches. CI were calculated using the t-distribution.

Product	Batch	Δ	Mean Δ	95% CI (lower)	95% CI (upper)	Δmax
Crispy Lettuce	1	1.05	1.72	0.12	3.31	2.33
	2	2.33				
	3	1.77				
Mix salad	1	3.62	3.61	3.48	3.73	3.65
	2	3.55				
	3	3.65				
Mâche (A)	1	0	0.16	0	0.83	0.47
	2	0				
	3	0.47				
Mâche (B)	1	0	0.07	0	0.37	0.21
	2	0				
	3	0.21				
Baby Lettuce	1	1.18	1.99	0.18	3.8	2.6
	2	2.19				
	3	2.6				
