# Supplementary material for: Safety of Ready-to-Eat Green Leafy Salads: Growth Potential of Listeria monocytogenes During Shelf Life
Source: Foods. 2026 Mar 25;15(7):1136. doi: 10.3390/foods15071136 (PMC13072739; doi:10.3390/foods15071136)
Supplement: Supplementary file 1 [file foods-15-01136-s001.zip › Supplementary_Table_S2_ANOVA.pdf]

Supplementary Table S2. One-way ANOVA of growth potential (Δ) across five RTE salad products.
The table reports degrees of freedom (Df), sum of squares (Sum Sq), mean squares (Mean Sq), F value, and p-value for the ANOVA model. A significant effect of product on growth potential was observed ($F(4,10) = 31.3$, $p < 0.05$).

Source	Df	Sum Sq	Mean Sq	F value	p-value
Product	4	25.742	6.436	31.3	1.25e-05
Residuals	10	2.056	0.206		
