# Supplementary material for: Safety of Ready-to-Eat Green Leafy Salads: Growth Potential of Listeria monocytogenes During Shelf Life
Source: Foods. 2026 Mar 25;15(7):1136. doi: 10.3390/foods15071136 (PMC13072739; doi:10.3390/foods15071136)
Supplement: Supplementary file 1 [file foods-15-01136-s001.zip › Supplementary_Table_S3_Tukey.pdf]

Supplementary Table S3. Pairwise comparisons of growth potential (Δ) among five RTE salad products using Tukey HSD.

The table reports mean differences (Δ) between products, 95% confidence intervals (CI) for the differences, and adjusted p-values for multiple comparisons. Comparisons with adjusted $p < 0.05$ indicate statistically significant differences in growth potential.

Comparison	Mean Difference (Δ)	95% CI Lower	95% CI Upper	Adjusted p-value
Crispy Lettuce – Baby Lettuce	-0.269	-1.487	0.95	0.945
Mâche (A) – Baby Lettuce	-1.832	-3.051	-0.614	0.004
Mâche (B) – Baby Lettuce	-1.918	-3.136	-0.699	0.003
Mix salad – Baby Lettuce	1.62	0.402	2.838	0.009
Mâche (A) – Crispy Lettuce	-1.563	-2.782	-0.345	0.012
Mâche (B) – Crispy Lettuce	-1.649	-2.867	-0.43	0.008
Mix salad – Crispy Lettuce	1.889	0.67	3.107	0.003
Mâche (B) – Mâche (A)	-0.086	-1.304	1.133	0.999
Mix salad – Mâche (A)	3.452	2.234	4.671	0.000023
Mix salad – Mâche (B)	3.538	2.319	4.756	0.000019
